# A Novel *LAMA2* Mutation (c.7412G>A) Was Found in a Chinese Patient With Congenital Muscular Dystrophy

**DOI:** 10.1111/jcmm.70667

**Published:** 2025-08-01

**Authors:** Meifang Zhao, Yuxing Liu, Liangliang Fan, Zhaochuan Liu, Yao Deng, Lihong Tao

**Affiliations:** ^1^ Department of Nephrology, Xiangya Hospital Central South University Changsha China; ^2^ Department of Cell Biology, School of Life Sciences Central South University Changsha China; ^3^ Department of Clinical Medicine Xinjiang Medical University Urumqi China; ^4^ Department of Cardiovascular Surgery, National Clinical Research Center for Geriatric Disorders, Xiangya Hospital Central South University Changsha China; ^5^ Department of Neurology The Affiliated Hospital of Yangzhou University Yangzhou Jiangsu China

**Keywords:** *LAMA2*, MDC1A, mutation, whole‐exome sequencing

## Abstract

Congenital muscular dystrophy (CMD) is a genetic muscle disorder characterised by muscle weakness and degeneration, either present at birth or emerging in middle age, often leading to progressive disability. MDC1A is a subtype of CMD caused by mutations in the *LAMA2* gene. In this study, we investigated a family affected by CMD from a remote rural area. The proband exhibited typical muscle weakness symptoms, though with a delayed onset. By combining whole‐exome sequencing with bioinformatics analysis, we explored the genetic aetiology of this family. A novel homozygous missense mutation (NM_000426: c.7412G>A; p.G2471D) of the *LAMA2* gene was detected in the proband. The proband's parents were found to carry the heterozygous mutation. Bioinformatic analysis indicated that the amino acid residue is highly conserved and has low tolerance to variation, suggesting a high pathogenic potential of the mutation. Based on genetic analysis, the proband was subsequently diagnosed with MDC1A. In conclusion, a novel *LAMA2* mutation was identified in a Chinese family with CMD. This discovery not only offers valuable insights for the patient's diagnosis and potential therapeutic strategies but also broadens the known spectrum of *LAMA2* mutations.

## 1 Introduction

Congenital muscular dystrophy (CMD) is a group of inherited muscle diseases that exhibit significant heterogeneity in both clinical symptoms and genetic characteristics [1]. Typically, CMD is inherited in an autosomal recessive manner [2]. Although the precise incidence of CMD is uncertain, existing data indicate an incidence of approximately 0.563 cases per 100,000 in Italy and approximately 0.89 cases per 100,000 in North England. Studies in China have reported incidences ranging from 0.017 to 0.083 cases per 100,000 people [3, 4]. CMD is characterised by significant muscle weakness, with onset ranging from childhood to adulthood. In children, it typically presents as flaccid muscles, low tone, poor spontaneous movement, delayed gross motor development and joint or spinal stiffness [5, 6, 7]. In adults, symptoms include partial muscle weakness and fatigue, with worsening of symptoms with activity [8, 9]. Additionally, CMD can present with various complex phenotypes, which can be categorised into several distinct subtypes on the basis of their characteristics as follows: merosin‐deficient CMD type 1A (MDC1A), merosin‐deficient CMD type 1B (with muscle hypertrophy and respiratory failure) (MDC1B), merosin‐deficient congenital muscular dystrophy type 1C (with muscle hypertrophy) (MDC1C), merosin‐deficient congenital muscular dystrophy type 1D (with intellectual disability and abnormal glycosylation) (MDC1D) and CMD with cardiomyopathy, etc. [10, 11, 12, 13].

CMD is a genetically diverse muscle disorder with multiple causative genes, each of which plays a distinct role in the pathogenesis of CMD [14]. Different genes contribute to disease development in various ways. The laminin protein encoded by the *LAMA2* gene plays a crucial role in maintaining the connection between muscle fibres and the surrounding matrix [15]. Mutations in the *LAMA2* gene can lead to impaired connections between muscle fibres and the matrix, affecting muscle function [16]. Collagen VI, encoded by the *COL6A1*, *COL6A2* and *COL6A3* genes, is an essential component of muscle and other connective tissues [17]. Mutations in these genes can impair collagen function, affecting the stability and strength of muscles. Alpha‐dystroglycan (α‐DG) maintains the integrity and stability of muscle cells by connecting to the extracellular matrix and the intracellular cytoskeleton, and its function depends on its glycosylation status [18]. Proteins responsible for the glycosylation of α‐DG, such as those encoded by *FKRP*, *FKTN*, *POMT1*, *POMT2*, *LARGE1*, *POMGNT1* and *ISPD*, are essential for this process [19]. Mutations in these genes can lead to defects in α‐DG glycosylation, affecting its connection function with the extracellular matrix and causing impaired stability of the muscle cell membrane [20]. Endoplasmic reticulum proteins, such as selenoprotein N, which is encoded by the *SELENON* gene, serve multiple functions within muscle cells, including protein folding and modification [21]. Defects in endoplasmic reticulum proteins can disrupt these processes, potentially leading to muscle diseases. Nuclear envelope proteins, such as lamins A/C, which are encoded by the *LMNA* gene, play a key role in maintaining the structure and function of the cell nucleus [22]. Defects in nuclear envelope proteins affect the nuclear function and overall structure of muscle cells and may lead to CMD. Accurate CMD diagnosis often requires a combination of clinical assessment, genetic testing and sometimes muscle biopsy to identify the specific gene mutation and subtype of CMD.

In the present study, we investigated a family from a remote rural area that has been affected by CMD. The proband exhibited typical muscle weakness symptoms, although with a delayed onset. By combining whole‐exome sequencing (WES) with bioinformatics analysis, we explored the genetic aetiology of this family.

## 2 Materials and Methods

### 2.1 Participants and Ethical Approval

A family from China affected by CMD presented at our hospital. The proband exhibited significant symptoms of muscle weakness, including difficulty turning over and a weak grasp. We gathered information about the proband's family history and conducted relevant examinations. Blood samples were collected from all family members for research purposes. To exclude polymorphisms, 200 healthy subjects, as described in our previous study, were also included in the study [23].

This study was approved by the Ethics Committee of The Affiliated Hospital of Yangzhou University (acceptance number is 2022‐YKL02‐G008). Written informed consent was obtained from all the subjects who participated in this study. Patients or the public were not involved in the design, conduct, reporting or dissemination plans of our research.

### 2.2 Immunohistochemical Staining

The tissue samples obtained from the participants were fixed in 4% paraformaldehyde and embedded in paraffin. The primary antibody was incubated overnight, and the secondary antibody was incubated for 1.5 h. The slides were washed with PBS, DAPI was added for nuclear staining, and an anti‐fluorescence quencher was added for observation. The following antibodies were used: CD3 (Abcam, ab237721), CD4 (Abcam, ab183685), CD8 (Abcam, ab217344), CD20 (Abcam, ab64088) and CD68 (ProMab Biotechnologies Inc., 30,621).

Haematoxylin–eosin (H&E) staining and oil red O (ORO), nicotinamide adenine dinucleotide hydride (NADH‐TR), membrane attack complex (MAC), histocompatibility complex (MHC)‐1 and modified Gomori tricolour (MGT) staining were completed by the clinical laboratory of the hospital, and magnetic resonance imaging (MRI) was performed by the imaging department of the hospital. We carefully recorded the results of these evaluations.

### 2.3 WES

Genomic DNA was extracted from the peripheral blood cells of all participants using the DNeasy Blood & Tissue Kit (Qiagen, Valencia, CA, USA), with the core components of the WES service and bioinformatics analysis provided by the Novogene Bioinformatics Institute (Beijing, China). Exons were captured using a SureSelect Human All Exon V6 Kit (Agilent, Santa Clara, CA, USA) and sequenced using a HiSeq X‐10 system (Illumina, San Diego, CA, USA). The strategies for data filtering were based on published literature and research findings from our laboratory [24, 25].

### 2.4 Mutation Validation and Cosegregation Analysis

Sanger sequencing was applied to confirm the candidate variants detected through WES. A segregation analysis was conducted among the relatives of the patient who participated in this research. The primer pairs (Table 1) were generated using the Integrated DNA Technologies PrimerQuest Tool, which can be accessed at the following website: http://sg.idtdna.com/Primerquest/Home/Index. The sequences of the PCR amplicons were deciphered utilising the ABI 3100 Genetic Analyser, a product of ABI located in Foster City, California.

**TABLE 1:** Design Sanger sequencing primers for the candidate genes.

Gene	F	R
*FMO5*	GGTGAAAGTGAAAGGAAATGTG	TCCAGAAACGGAAAGTCAAAG
*LAMA2*	GTGCAAGTGCTTGAGAAAGTC	CTTTCTGCAACCAAGGGTTATATG
*WWP1*	GGATTCAACAGACAGGGTTTAC	CAAGACGACTTAAGGCTGC
*SLC18A1*	TGTAAAGGCCATCGGTTTTC	GTGCTCCTTACAAGCTTCTC
*ATXN2*	GCAGCTCCTCGGAGTCCC	CATGGTGAGGGGCCCATAC
*ATP10A*	TGGTTTCTTTCTGCTCCGC	TTGGTGCTCTGGGTTCTGTG

### 2.5 Bioinformatics Analysis

Tolerance analysis was conducted using the MetaDome website (https://stuart.radboudumc.nl/metadome/dashboard). Conservation analysis was carried out with the ConSurf server (https://consurf.tau.ac.il/), and mutant modelling was performed using SWISS‐MODEL (https://swissmodel.expasy.org/interactive).

## 3 Results

### 3.1 Clinical Findings

**FIGURE 1:**
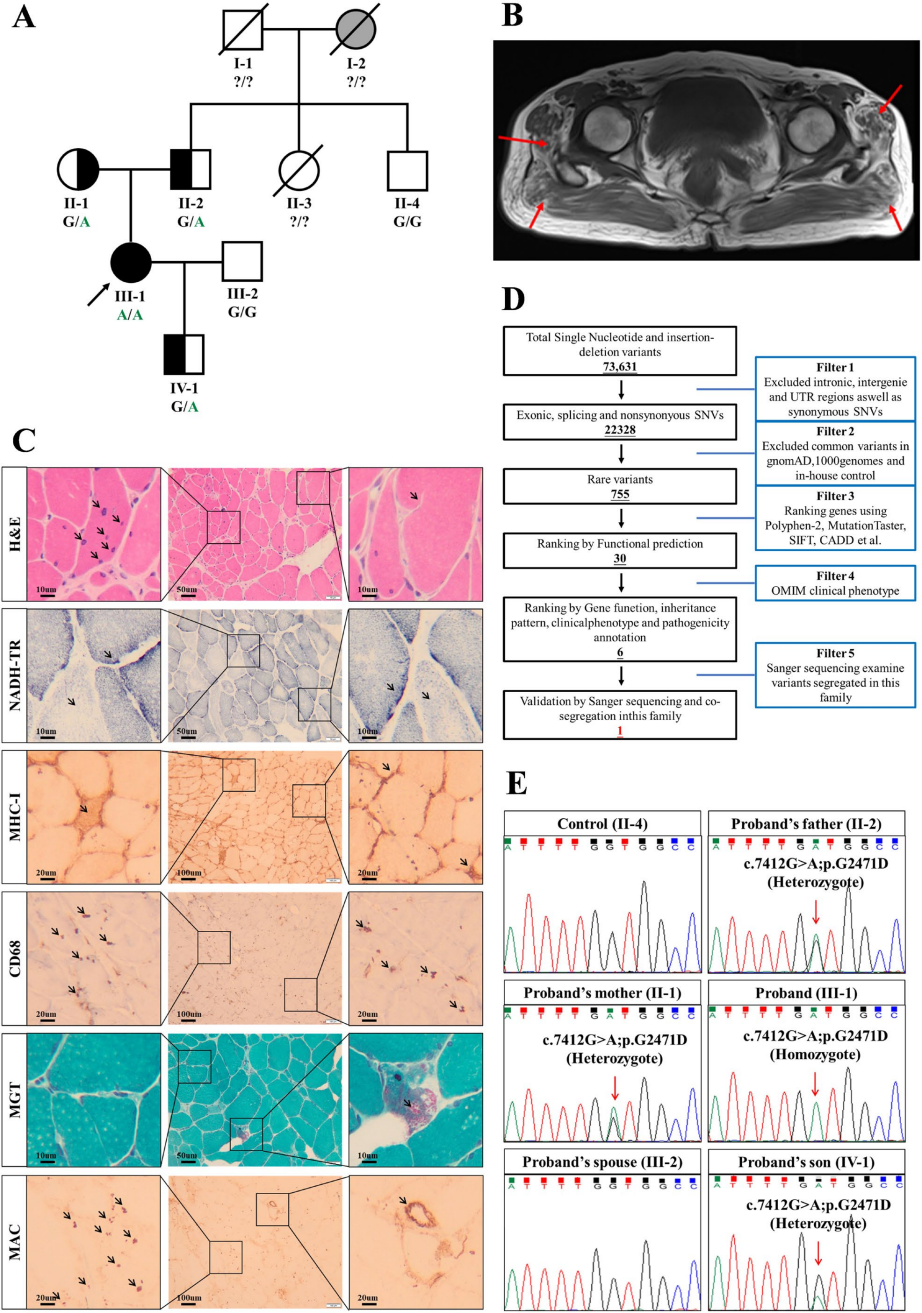
The proband's family and her clinical findings. (A) The genealogy of this CMD family. Squares represent the males; circles, females; black symbols, affected individuals; white symbols, unaffected individuals; arrow, the proband. (B) The MRI of pelvis: T1 sequence showed that the hip muscle tissue was replaced by adipose tissue, and the muscle was atrophy (red arrow). (C) The H&E staining revealed significant variation in muscle fibre size, with small fibres appearing as small circles and polygons. Thickened and split muscle fibres were also observed, while the red arrows denote nuclear migration and split muscle fibres; NADH staining reveals uneven distribution of oxidative enzyme activity within muscle fibres; Immunohistochemical staining for MHC‐I revealed that MHC protein was extensively expressed in the sarcolemma and cytoplasm of muscle fibres; CD68 immunostaining showed scattered expression of CD68 protein within muscle fibres; MGT staining clearly shows a red area, indicating the presence of ragged red fibres; Immunohistochemical analysis with MAC indicates aggregation in blood vessels and cytoplasm, suggesting myositis. (D) Iterative filtering identified 6 candidate mutations in 6 genes. (E) Analysis and identification of *LAMA2* gene mutation in proband and her parents and healthy controls. The homozygous missense mutation (c.7412G>A) was observed in the proband.

The proband (III‐1) was a 50‐year‐old woman from a remote rural area in South China (Figure 1A). She began experiencing muscle weakness 20 years prior, with symptoms progressively worsening over the years. In recent years, her muscle weakness had significantly intensified, making it difficult for her to grasp objects and coordinate her fingers. She also suffered from lumbar and back muscle weakness, which hampered her ability to turn over and stand, greatly affecting her daily life, and leading her to seek medical attention at our hospital. The proband initially underwent MRI scans of the skull, cervical spine and pelvis. The pelvic MRI scan revealed fatty infiltration of the pelvic girdle muscles, indicating muscle atrophy (Figure 1B). Cervical MRI scans revealed degenerative changes, whereas head MRI scans revealed no significant abnormalities (Figure S1A,B). Histological examination of the patient's tissue biopsy revealed myofiber degeneration, atrophy and fragmentation, along with muscle inflammation, which was consistent with a dystrophic phenotype. H&E staining revealed uneven muscle fibres, with some fibres appearing rounded and exhibiting nuclear internalisation, whereas others displayed signs of splitting. MGT staining revealed the presence of ragged red fibres in the muscle tissue. NADH‐RT staining revealed an uneven distribution and activity of oxidases within the muscle fibres. Immunohistochemistry for MHC‐I demonstrated widespread expression of MHC proteins in muscle fibre cell membranes and the cytoplasm. CD68 immunohistochemistry revealed scattered expression of CD68 within the muscle fibres. MAC staining revealed deposition in blood vessels and the cytoplasm, suggesting myositis (Figure 1C). Additionally, immunohistochemical tests for CD3, CD4, CD8 and CD20 were conducted on the muscle tissue, and no significant abnormalities were observed. ORO staining of the muscle tissue sections also revealed no significant increase in lipid droplets (Figure S1C). The needle electromyography results for the proband are shown in Table 2, indicating that nerve conduction was normal. Laboratory results of various serum levels measured in the proband are presented in Table 3. Notably, the proband's creatine kinase level was 1045.3 U/L, approximately 10 times higher than the normal range for women (25–200 U/L).

**TABLE 2:** Needle EMG results of the proband.

Side	Muscle	Insertional activity	Spontaneous activity			MUAP			Recruitment
			Fib	PSW	Fas	Dur	Amp	Poly	
Left	Extensor digitorum brevis	Normal	Normal	Normal	Normal	Normal	Normal	Irregular waves	Myopathic interference
Right	Paraspinal muscle L5	Normal	Normal	Normal	Normal	Normal	Normal	None	NA
Right	Deltoid	Normal	Normal	Normal	Normal	Normal	Normal	Irregular waves	Myopathic interference
Right	Abductor digiti minimi	Increase	Normal	1+	Normal	Normal	Normal	Irregular waves	Myopathic interference
Right	Abductor pollicis brevis	Increase	Normal	2+	Normal	Normal	Normal	None	Normal
Right	Dorsal interossei	Normal	Normal	Normal	Normal	Normal	Normal	None	NA
Right	Iliopsoas	Normal	Normal	Normal	Normal	Normal	Normal	Irregular waves	Myopathic interference
Right	Vastus medialis	Normal	Normal	Normal	Normal	Normal	Normal	None	Myopathic interference
Left	Vastus medialis	Normal	Normal	Normal	Normal	Normal	Normal	Irregular waves	Myopathic interference
Right	Tibialis anterior	Increase	Normal	1+	Normal	Normal	Normal	None	Myopathic interference
Right	Extensor digitorum brevis	Normal	Normal	1+	Normal	Normal	Normal	None	Normal
Left	Gastrocnemius	Increase	Normal	2+	Normal	Normal	Normal	None	Normal

Abbreviations: Amp, amplitude; Dur, duration; Fas, fasciculation; Fib, fibrillation; NA, not available; Poly, polyphasics; PSW, positive sharp waves.

**TABLE 3:** Results of serum and laboratory investigations in various measurements in the proband.

Laboratory values	Laboratory values	Normal values (range)
TC (mmol/L)	4.19	2.86–5.98
TG (mmol/L)	2.58 ↑	0.56–1.70
HDL‐C (mmol/L)	1.04 ↓	1.05–1.55
LDL‐C (mmol/L)	1.83 ↓	2.07–3.37
CK (U/L)	1045.3 ↑	25–200
CK‐MB (U/L)	27.8 ↑	0–18
LDH (U/L)	190.0	50–240
AST (U/L)	26.1	5–40
HBDB (U/L)	128.4	80–220
Na+ (mmol/L)	139	137–147
Mg2+ (mmol/L)	0.84	0.75–1.02
Cl‐ (mmol/L)	103	99–110
K+ (mmol/L)	3.30 ↓	3.5–5.3
Ca2+ (mmol/L)	2.30	2.11–2.52

Abbreviations: AST, aspartate aminotransferase; Ca2+, calcium; CK, creatine kinase; CK‐MB, creatine kinase isoenzyme MB; Cl‐, chloride; HBDB, α‐hydroxybutyrate dehydrogenase; HDL‐C, high‐density lipoprotein cholesterol; K+, potassium; LDH, lactate dehydrogenase; LDL‐C, low‐density lipoprotein cholesterol; Mg2+, magnesium; Na+, sodium; TC, total cholesterol; TG, triglycerides.

The proband's family history revealed that both paternal grandparents were deceased. The grandmother (I‐2) had symptoms of lower limb muscle weakness and difficulty standing, whereas the grandfather (I‐1) showed no evidence of muscle weakness. The proband's paternal aunt passed away in an accident, with no further details available. The proband's parents, paternal uncle, spouse and son all denied any symptoms related to muscle weakness.

### 3.2 Genetic Analysis Results

**FIGURE 2:**
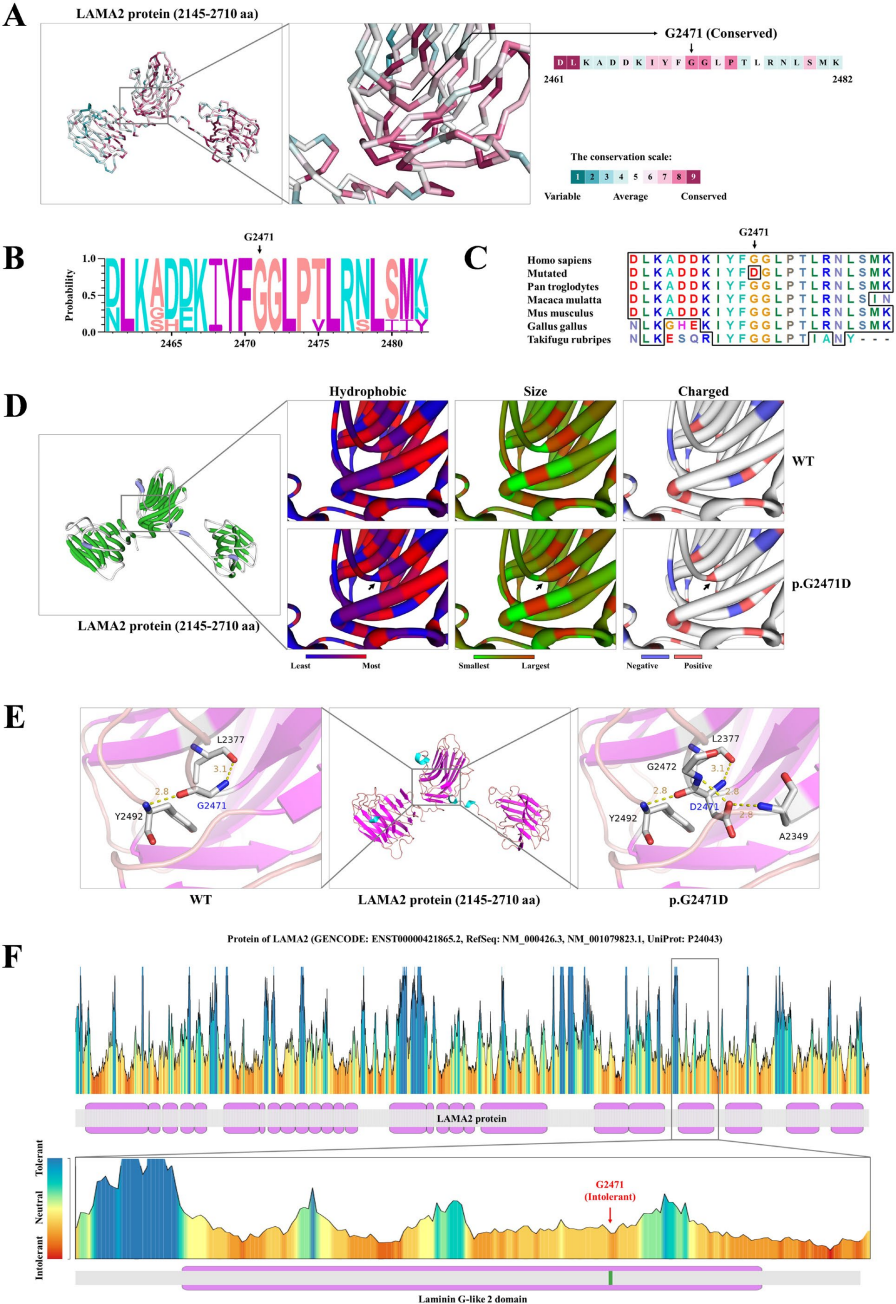
Analysis of protein function changes following genetic mutations. (A) The analysis examined the changes in hydrophobicity, size and charge before and after the amino acid alteration. (B) The analysis assessed the changes in hydrogen bonding patterns before and after the amino acid alteration. (C) A conservation analysis of the LAMA2 protein's amino acid sequence revealed that the mutation site has high conservation. (D) Base sequence analysis of the *LAMA2* gene visualised through a diagram shows that the mutation site has high base conservation. (E) Comparative analysis of the LAMA2 amino acid sequence across multiple species reveals high conservation at the mutation site. (F) An analysis of the tolerance of the LAMA2 protein's amino acid sequence indicates that the mutation site is located in an intolerant region.

WES generated 12 Gb of data, with a coverage rate of 97.94% in the target region and a coverage rate of 99.2% for the target region above 10×. After alignment and single‐nucleotide variant calling, 74,251 variants were identified in the proband. Data filtering was performed as shown in Figure 1D. After the data were filtered, a set of six possible pathogenic variants in six genes was identified in the proband (Table 4). After Sanger sequencing validation and genotype–phenotype cosegregation analysis, a homozygous missense mutation (NM_000426: c.7412G>A;p.G2471D) of the *LAMA2* gene was identified in the proband. The proband's parents (II‐1 and II‐2) and son (IV‐1) all carried a heterozygous mutation at this locus. The proband's uncle (II‐4) and spouse (III‐2) did not carry the mutation (Figure 1A,E). This mutation was not found in our cohort of 200 healthy controls. The mutation (c.7412G>A; p.G2471D) is located in exon 52 of the *LAMA2* gene, resulting in the substitution of the glycine (G) residue at position 2471 of the LAMA2 protein with aspartic acid (D). ConSurf server software predicted that the G2471 amino acid is located in a conserved region of the LAMA2 protein (Figure 2A). Similarly, multiple sequence alignment of LAMA2 amino acid sequences across various species confirmed that the G2471 position is highly conserved (Figure 2B,C). Using SWISS‐MODEL software, modelling was performed for both the normal and mutated proteins. Comparisons revealed that the mutation altered the hydrophobicity, size and polarity of the modified residue (Figure 2D). Additionally, ExPASy server software predicted that the hydrophilicity, hydrophobicity, polarity and molecular weight of the amino acid residues near G2471 would change upon mutation (Figure S2A–D). Additionally, this mutation may alter the hydrogen bonding interactions between the G2471 amino acid residue of the LAMA protein and surrounding residues, as illustrated by the dashed lines in Figure 2E. Furthermore, MetaDome software predicted the G2471 amino acid is located in the intolerant region of the LAMA2 protein (Figure 2F). Therefore, we identified the *LAMA2* mutation as disease causing.

**TABLE 4:** Pathogenicity prediction of the six possible pathogenic mutations was made by different bioinformatic prediction methods.

Gene	Amino acids change	1000g2015aug	ExAC	gnomAD exome	SIFT	POLYPHEN‐2	Mutation taster	ACMG statement
*FMO5*	NM_001144829; c.961G>A; p.D321N	—	—	0.00000398737	Damaging (0.019)	Probably damaging (0.794)	Disease causing (1)	PM1 + PM2
*LAMA2*	NM_000426; c.7412G>A; p.G2471D	—	—	0.00000399517	Damaging (0.0)	Probably damaging (1.0)	Disease causing (1)	PM1 + PM2 + PP3
*WWP1*	NM_007013; c.1463G>A; p.R488K	—	—	—	Damaging (0.002)	Probably damaging (1.0)	Disease causing (1)	PM1 + PM2
*SLC18A1*	NM_001142324; c.1337 T>C; p.L446P	—	—	0.00000404269	Damaging (0.0)	Probably damaging (0.997)	Disease causing (1)	PM1 + PM2 + PP3
*ATXN2*	NM_002973; c.295G>A; p.G99R	—	—	—	Damaging (0.0)	Probably damaging (1.0)	Disease causing (1)	PM2
*ATP10A*	NM_024490; c.1369C>A; p.R457S	—	—	—	Damaging (0.035)	Probably damaging (0.951)	Disease causing (1)	PM1 + PM2

## 4 Discussion

In our study, we investigated a family from a remote rural area that was affected by CMD. The proband exhibited typical muscle weakness symptoms, although with a delayed onset. Through WES, a homozygote mutation (c.7412G>A; p.G2471D) in the *LAMA2* gene was identified in the proband. Sanger analysis revealed that the proband's parents (II‐1 and II‐2) and son (IV‐1) were both heterozygous carriers of the mutation. The proband's mother (II‐1) had diabetes and hypertension, and her father (II‐2) suffered from gout due to long‐term alcohol abuse, but both denied having any muscle weakness symptoms. Although the proband described that her grandmother (I‐2) had symptoms of lower limb muscle weakness and difficulty standing before she did, it was not possible to obtain a blood sample to confirm whether the proband's father's (II‐2) mutation was inherited from her grandmother (I‐2). Owing to the passage of time, no valuable family history information could be obtained from the maternal side. The proband's son (IV‐1) also denied any muscle weakness and was currently unmarried. We recommended genetic screening when the patient plans to marry in the future.

On the basis of the genetic testing results, the proband in this family was further molecularly diagnosed with MDC1A. In most reported cases of MDC1A, only the H&E staining results of pathological muscle sections are typically described, showing muscle fibre hypertrophy, fragmentation and inflammatory cell infiltration, without further differentiation of inflammatory cell types or in‐depth analysis of inflammation‐related protein expression [26]. In contrast, we performed staining for various cell surface antigen markers on the proband's muscle sections, along with an additional lipid deposition analysis. The immunohistochemical staining results for MHC‐I and CD68 in the proband revealed high expression. The MHC‐I protein activates immune cells through antigen presentation, inducing the expression of proteins such as CD68 [27, 28]. The high expression of CD68 suggests severe muscle inflammation, which may be a key factor in triggering autoimmune responses and pain and was likely a significant source of patient discomfort. However, in our study, no significant changes were observed in CD3, CD4, CD8 or CD20 expression, and no increase in lipid droplets was detected by Oil Red O staining, suggesting that the mutation may not affect muscle tissue‐related immunity or lipid metabolism. There is no effective treatment currently available for MDC1A [29, 30]. Therefore, management focuses on symptomatic treatment for patients. Potassium chloride oral solution was prescribed to alleviate muscle weakness caused by hypokalaemia. Additionally, corticosteroid therapy with oral prednisolone acetate was employed to reduce muscle inflammation [31]. During treatment, a low‐salt and low‐sodium diet was recommended, along with adequate rest to avoid fatigue and prevent the exacerbation of symptoms [32, 33]. The proband reported some relief from muscle weakness after starting the medication. At the six‐month follow‐up, the patient continued to experience muscle weakness; however, there was no worsening of symptoms.

Most patients with *LAMA2* gene mutations present symptoms during childhood. For example, a Colombian girl carrying the mutations c.4198C>T and c.9227_9243dup was diagnosed with peripheral hypotonia at just two months of age, with symptoms progressively worsening over the years [34]. In another report from Morocco, a girl with a homozygous (c.2217G>A; p.Trp739*) mutation presented with delayed motor development and tetraplegia at the age of one [35]. Additionally, a 13‐year‐old patient with a clinical history dating back to 18 months of age experienced worsening symptoms annually, and genetic sequencing revealed a homozygous *LAMA2* mutation (c.1854_1861dup; p.Leu621Hisfs*7) [36]. However, a minority of patients begin to show symptoms only during young adulthood. For example, a Japanese patient was diagnosed with mild myopathy and diffuse muscle atrophy at the age of 26, with genetic testing revealing a homozygous mutation in the *LAMA2* gene (c.818G>A; p.Arg273Lys) [37]. In another case, a 28‐year‐old Indian man experienced gradually progressive weakness in the lower limbs, primarily proximal, over the past three years, with elevated creatine phosphokinase levels and brain MRI showing diffuse symmetric periventricular white matter hyperintensities. WES revealed a homozygous missense variant in exon 4 of the *LAMA2* gene (c.442C>T; p.Arg148Trp) [38]. The patient in our report represents a relatively rare late‐onset form of the disease, offering valuable new insights for research into this condition.

**FIGURE 3:**
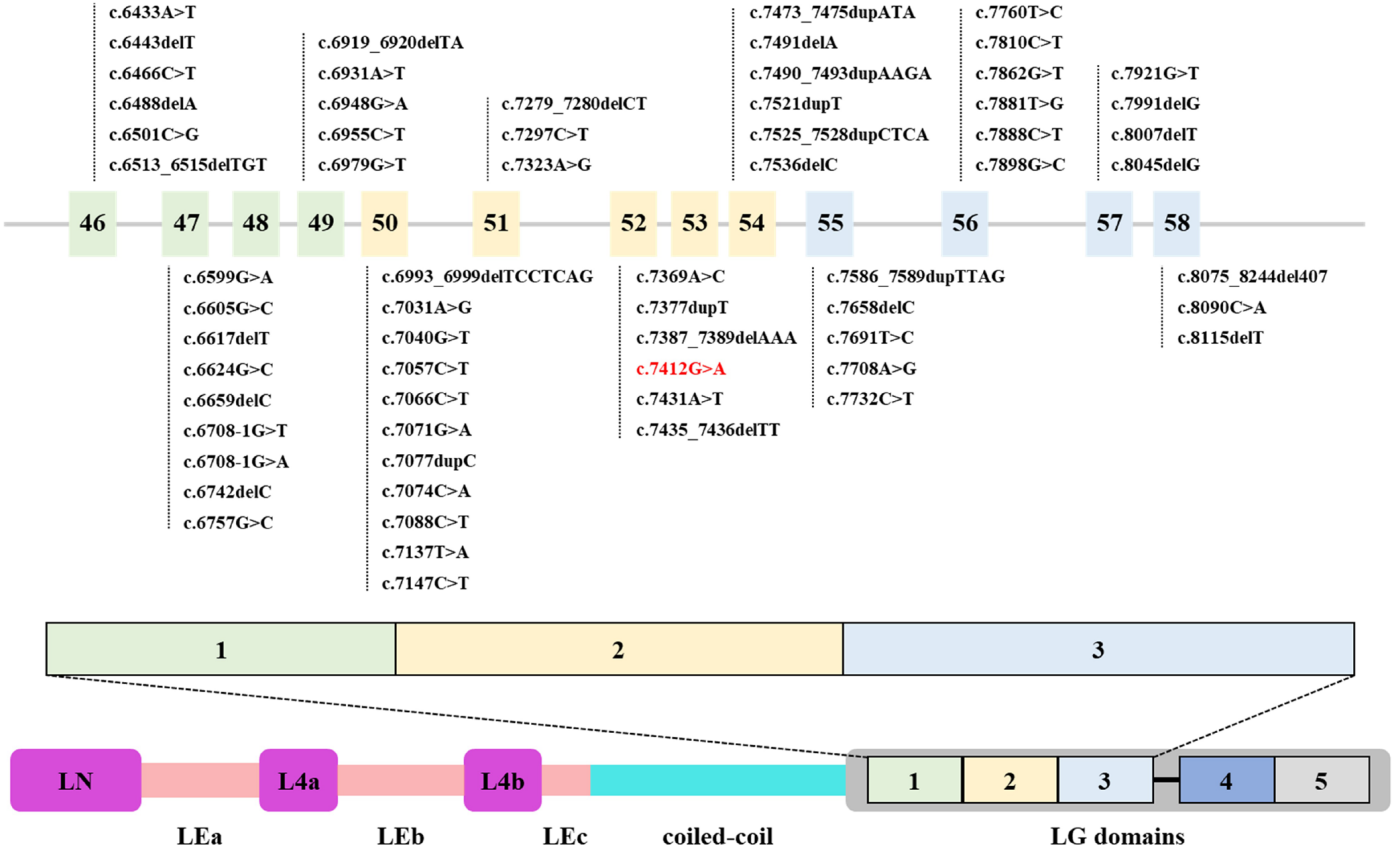
LAMA2 protein domain and LG1‐3 domain mutation summary figure. The LAMA2 protein is primarily composed of LN domains and LG domains, which can be functionally subdivided into LG1‐3 and LG4‐5. This chart summarises mutations occurring in the LG1‐3 region. Exons within the same protein domain are indicated by boxes of the same colour, and mutations reported in this study are highlighted in red.

Laminin, a key extracellular protein in the basement membrane, facilitates cell attachment, migration and tissue organisation during embryonic development through interactions with the extracellular matrix [33]. It consists of three subunits, namely, alpha, beta and gamma, which are linked by disulphide bonds in a cross‐shaped structure. The *LAMA2* gene encodes the laminin‐α2 chain, a component of laminin 2 (merosin) and laminin 4 (s‐merosin) [33, 39]. Pathogenic variations in this gene have been identified as the cause of MDC1A, an autosomal recessive genetic disorder. The laminin‐α2 chain, a protein with a molecular weight of 380 kDa, encompasses two core structural domains, namely, the LN domain and the LG domain, which are essential for the formation of laminin [40]. The LG domain is further divided into five parts: LG1 to LG5. Specifically, LG1‐3 interacts with integrin α7β1, while LG4‐5 binds to α‐dystroglycan (α‐DG) and sulphated glycolipids in Schwann cell basement membranes (SC BMs) [41, 42, 43]. These interactions are crucial for anchoring the cell membrane and the underlying cytoskeleton. Specifically, the LG1‐3 structural domain is the primary binding site for integrins. Integrins are cell surface receptors that are responsible for linking the extracellular matrix (ECM) to the intracellular cytoskeleton. This binding is crucial for cell adhesion, migration and signal transduction. Through interaction with integrins, the LG1‐3 domain can activate various cellular signalling pathways, such as those involving FAK and Src family kinases, which are involved in regulating cell adhesion and migration, thereby affecting cell activity and function. Additionally, the LG1‐3 domain contributes to the assembly and maintenance of the basement membrane by binding to integrins. It promotes the accumulation of the LAMA2 protein on the cell surface, forming a stable basement membrane structure. Dysfunction of the LG1‐3 domain may lead to muscle weakness and atrophy, as it affects the adhesion and signal transduction between muscle cells and the basement membrane. Moreover, in peripheral nerves, abnormalities in the LG1‐3 domain may affect the myelination and function of Schwann cells, leading to slowed nerve conduction velocities and neuropathy. Abnormalities in the LG1‐3 domain can also lead to instability and defects in the basement membrane, affecting the integrity and function of tissues (31,920,536, 38,825,010, 31,782,895). In this study, we identified a homozygous mutation in the *LAMA2* gene within a CMD family, which is located in the LG2 domain where the LAMA2 protein performs important functions. The mutation results in the replacement of glycine at position 2471 with aspartic acid. Through bioinformatics analysis, we visualised the area surrounding this residue before and after the mutation, and the results revealed that after mutation, the physicochemical properties of this site, including hydrophobicity and charge, were altered, affecting the surrounding areas. Additionally, the hydrogen bonding strength between the amino acids at this site and the surrounding amino acids changed significantly. These changes could affect the structure and function of the protein, leading to the onset of the disease. In addition, the LG1‐3 region is highly conserved and a hotspot for pathogenic mutations. A compilation of all reported pathogenic mutations of the *LAMA2* gene that are located within the LG1‐3 structural domain of the LAMA2 protein is shown in Figure 3. This information may be helpful in the diagnosis and treatment of *LAMA2* mutation‐related diseases. Notably, the mutation reported in this study has not been previously reported in the literature, indicating that it is a novel pathogenic mutation of the *LAMA2* gene.

## 5 Conclusions

In conclusion, we identified a novel homozygous missense mutation (NM_000426: c.7412G>A; p.G2471D) of the *LAMA2* gene in a Chinese family with CMD. Our research not only provides a definitive diagnosis for patients but also offers guidance for genetic counselling within the family. Moreover, the findings expand the mutation spectrum of the *LAMA2* gene, offering valuable insights for future research in this field.

## Author Contributions

**Meifang Zhao:** software (equal), validation (equal), visualization (equal), writing – original draft (equal). **Yuxing Liu:** data curation (equal), formal analysis (equal). **Liangliang Fan:** investigation (equal), supervision (equal). **Zhaochuan Liu:** writing – original draft (supporting). **Yao Deng:** funding acquisition (equal), investigation (equal), writing – review and editing (equal). **Lihong Tao:** funding acquisition (equal), investigation (equal), project administration (equal), resources (equal), writing – review and editing (equal).

## Conflicts of Interest

The authors declare no conflicts of interest.

## Supporting information


**Figure S1.** Other clinical findings of the proband. (A) The MRI examination of the cervical spine revealed intervertebral disc herniation and degenerative changes in the patient, and given the patient's age, the disease is considered to have low correlation with genetic factors. (B) MRI examination of the head showed no abnormality. (C) Immunohistochemistry for CD3, CD4, CD8 and CD20 shows no significant abnormalities. Oil Red O staining reveals no obvious lipid deposition.

**Figure S2.** Physicochemical property analysis of the p.G2471D mutation site in the LAMA2 protein. This figure illustrates the physicochemical properties around the p.G2471D mutation site in the LAMA2 protein, including hydrophilicity (A), hydrophobicity (B), polarity (C) and molecular weight (D). The horizontal axis of the graph represents the amino acid sequence positions in the LAMA2 protein, while the vertical axis indicates different scores for the physicochemical properties. In graph A, the hydrophilicity score shows the changes in hydrophilicity of amino acid residues near the mutation site. A more negative score indicates stronger hydrophilicity, while a more positive score indicates stronger hydrophobicity. Graph B provides a hydrophobicity score, where a more negative score indicates stronger hydrophobicity, and a more positive score indicates stronger hydrophilicity. Graph C displays changes in polarity scores, with higher scores indicating stronger polarity. In graph D, the molecular weight score shows the variation in molecular weight of amino acid residues near the mutation site. A higher score indicates a larger molecular weight, while a lower score indicates a smaller molecular weight.

## Data Availability

The original contributions are included in the Supplementary material and the following database: GSE‐Human repository, accession number: HRA008580 (https://ngdc.cncb.ac.cn/gsa‐human/submit/hra/submit).
