# Supplementary figures and images for: A Novel LAMA2 Mutation (c.7412G>A) Was Found in a Chinese Patient With Congenital Muscular Dystrophy
Source: J Cell Mol Med. 2025 Aug 1;29(15):e70667. doi: 10.1111/jcmm.70667 (PMC12316597; doi:10.1111/jcmm.70667)

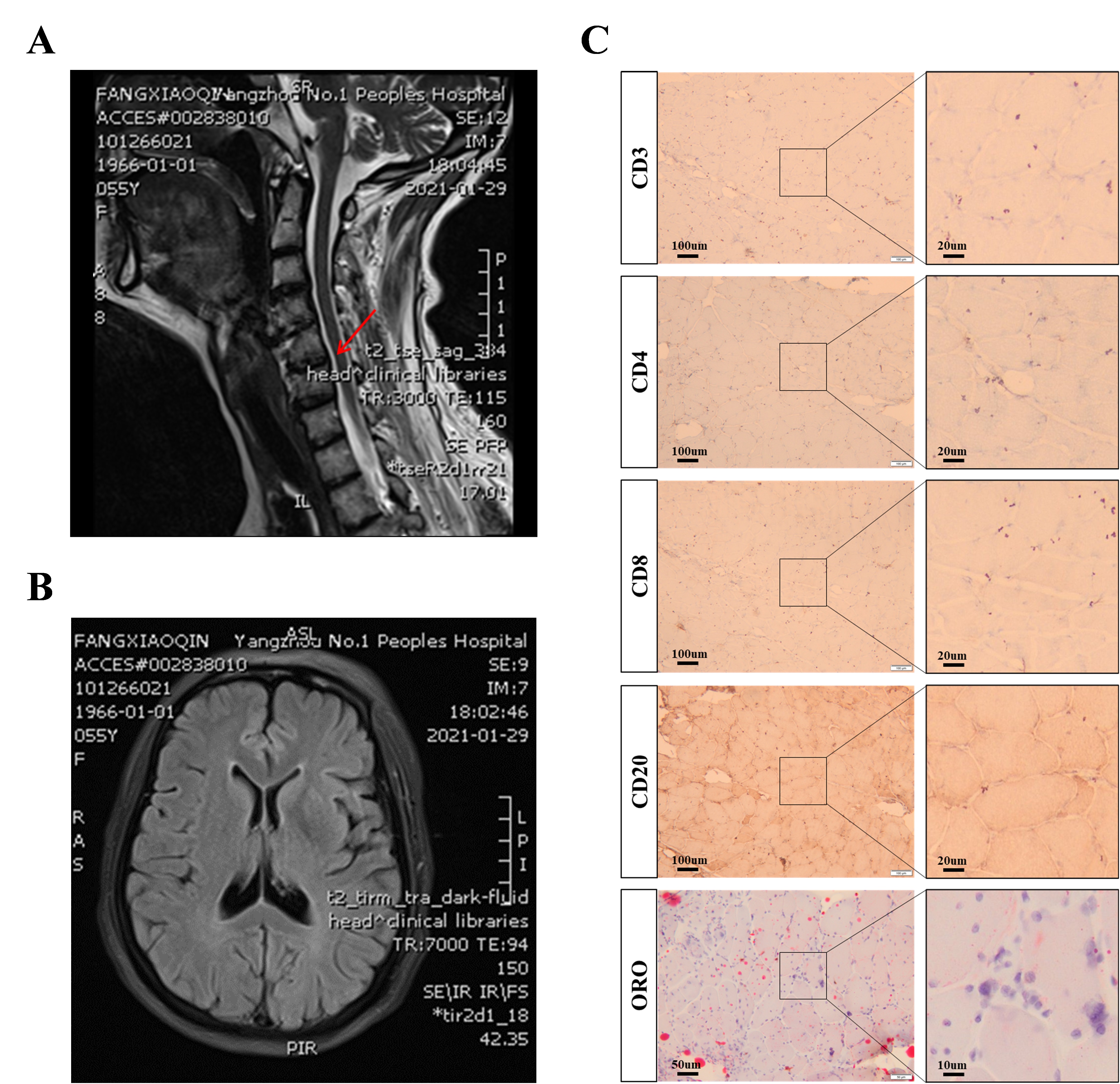

Supplement: Supplementary file 1 — Figure S1. Other clinical findings of the proband. (A) The MRI examination of the cervical spine revealed intervertebral disc herniation and degenerative changes in the patient, and given the patient's age, the disease is considered to have low correlation with genetic factors. (B) MRI examination of the head showed no abnormality. (C) Immunohistochemistry for CD3, CD4, CD8 and CD20 shows no significant abnormalities. Oil Red O staining reveals no obvious lipid deposition. [file JCMM-29-e70667-s001.jpg]

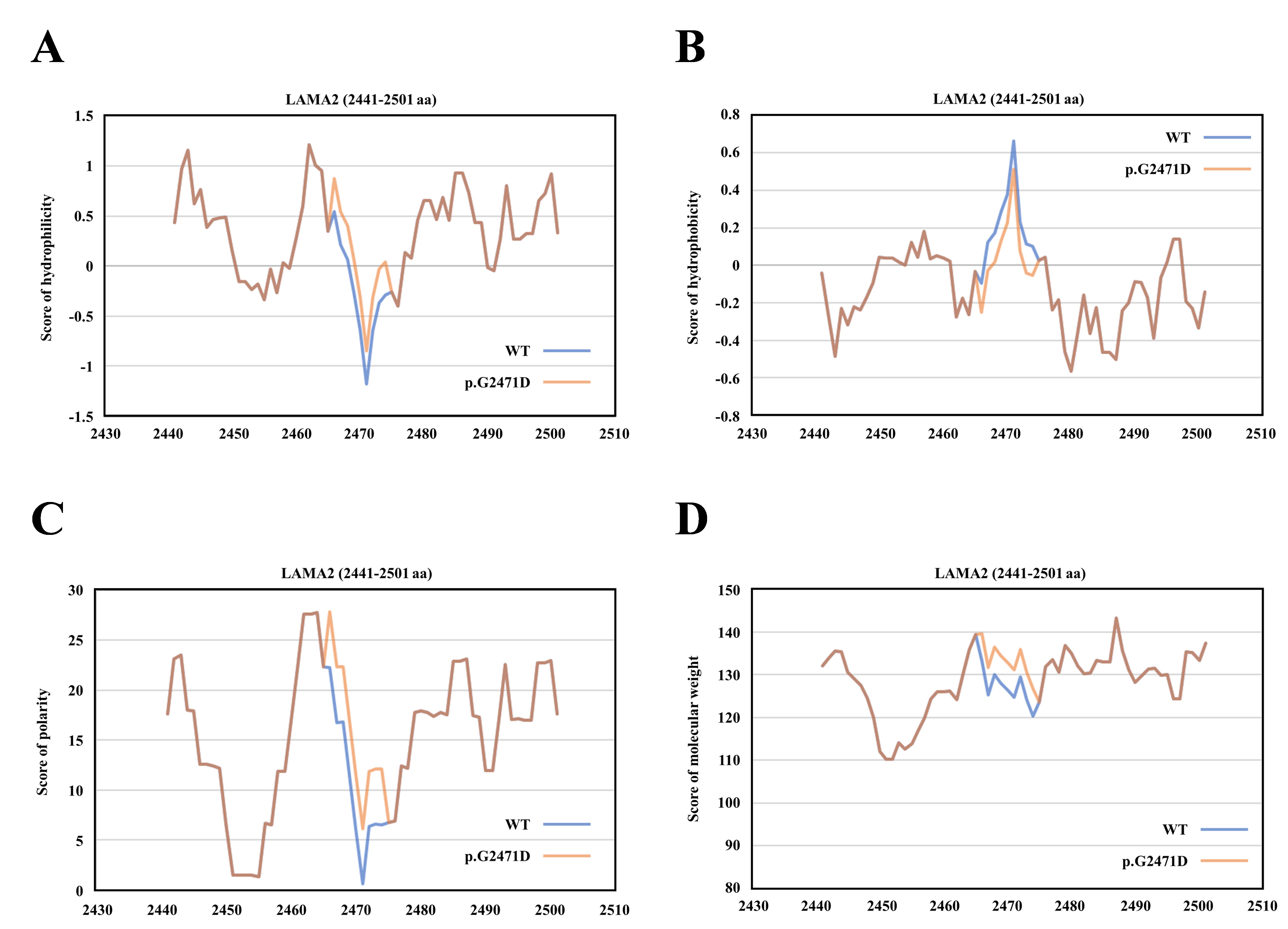

Supplement: Supplementary file 2 — Figure S2. Physicochemical property analysis of the p.G2471D mutation site in the LAMA2 protein. This figure illustrates the physicochemical properties around the p.G2471D mutation site in the LAMA2 protein, including hydrophilicity (A), hydrophobicity (B), polarity (C) and molecular weight (D). The horizontal axis of the graph represents the amino acid sequence positions in the LAMA2 protein, while the vertical axis indicates different scores for the physicochemical properties. In graph A, the hydrophilicity score shows the changes in hydrophilicity of amino acid residues near the mutation site. A more negative score indicates stronger hydrophilicity, while a more positive score indicates stronger hydrophobicity. Graph B provides a hydrophobicity score, where a more negative score indicates stronger hydrophobicity, and a more positive score indicates stronger hydrophilicity. Graph C displays changes in polarity scores, with higher scores indicating stronger polarity. In graph D, the molecular weight score shows the variation in molecular weight of amino acid residues near the mutation site. A higher score indicates a larger molecular weight, while a lower score indicates a smaller molecular weight. [file JCMM-29-e70667-s002.jpg]
